# Developing a Blockchain-Based Supply Chain System for Advanced Therapies: Protocol for a Feasibility Study

**DOI:** 10.2196/17005

**Published:** 2020-12-14

**Authors:** Ching Lam, Michelle Helena van Velthoven, Edward Meinert

**Affiliations:** 1 Digitally Enabled PrevenTative Health (DEPTH) Research Group Department of Paediatrics University of Oxford Oxford United Kingdom; 2 Department of Primary Care and Public Health Imperial College London London United Kingdom

**Keywords:** blockchain, digital health, IOT, internet of things, regenerative medicine

## Abstract

**Background:**

Advanced therapies, including cell and gene therapies, have shown therapeutic promise in curing life-threatening diseases, such as leukemia and lymphoma. However, these therapies can be complicated and expensive to deliver due to their sensitivity to environment; troublesome tissue, cell, or genetic material sourcing; and complicated regulatory requirements.

**Objective:**

This study aims to create a novel connected supply chain logistics and manufacturing management platform based on blockchain, with cell and gene therapy as a use case. Objectives are to define the requirements and perform feasibility evaluations on the use of blockchain for standardized manufacturing and establishment of a chain of custody for the needle-to-needle delivery of autologous cell and gene therapies. A way of lowering overall regulatory compliance costs for running a network of facilities operating similar or parallel processes will be evaluated by lowering the monitoring costs through publishing zero-knowledge proofs and product release by exception.

**Methods:**

The study will use blockchain technologies to digitally connect and integrate supply chain with manufacturing to address the security, scheduling, and communication issues between advanced therapy treatment centers and manufacturing facilities in order to realize a transparent, secure, automated, and cost-effective solution to the delivery of these life-saving therapies. An agile software development methodology will be used to develop, implement, and evaluate the system. The system will adhere to the EU and US good manufacturing practices and regulatory requirements.

**Results:**

This is a proposed study protocol, and upon acceptance, grant funding will be pursued for its execution in 2021.

**Conclusions:**

The successful implementation of the integrated blockchain solution to supply chain and manufacturing of advanced therapies can push the industry standards toward a safer and more secure therapy delivery process.

**International Registered Report Identifier (IRRID):**

PRR1-10.2196/17005

## Introduction

Advanced therapy medicinal products (ATMPs) industries, including cell therapy and gene therapy, are forecasted to grow at a compound annual growth rate of 22% from 2018 to 2022 [1]. ATMPs are estimated to be a billion-dollar industry and potentially the fourth therapeutic pillar of health care [2,3]. Commercially available ATMPs make up a very small percentage of total patient interventions as compared to the mainstream first- and second-line therapies in the developed markets. However, for cell and gene therapy, they are expected to grow from a few 1000 patients in 2018-2019 globally to possibly 100 million by 2025, as the number of commercially available ATMPs matures [1].

To facilitate the growth of the ATMP sector, the supply chain infrastructure is critical in supporting the industry from clinical trials to commercial distribution of life-saving treatments to more patients in a safer and more secure manner. In addition, compliance with data protection laws (Health Insurance Portability and Accountability Act [HIPAA] in the United States and General Data Protection Regulation [GDPR] in the European Union) comes at a very high cost [4-6]. Without proper encryption and selective data disclosure, everyone in the supply chain will have to be GDPR-compliant. Key to the success of these new treatments will be the safe, secure, and timely delivery of advanced therapies in a highly regulated environment (eg, temperature, tilt, time duration, and location) to ensure transparency and accuracy [7,8].

Current logistics tracking solutions are reliant on paper audit trails and legacy cloud infrastructure and are at significant risk of causing treatment failures. Specific to autologous therapies, the starting material collected from the patients can vary greatly. This can cause the final manufactured product to be of different quality, which may cause it to fall out of specification [9]. For example, Novartis had to write off multiple batches of Kymriah (Novartis) due to out-of-specification products since its launch in August 2017 in the United States [10]. Similarly, during the clinical studies, 7%-9% of patients did not receive the CAR-T treatment due to manufacturing failures [11].

Current solutions based on paper and dated legacy standalone information technology (IT) infrastructure that are being used by bioscience companies during precommercialization may be sufficient during research stages. However, these solutions are likely to fail as their operations scale up and out. They are not designed to be scalable, interoperable, or capable of dealing with the complexity of regulation and process that these ATMPs will incur in the real world of patient care. Batch identification through patient initials and date of birth is considered insufficient due to possible mix-ups, which can be catastrophic for patients. Intuitive labeling methods must be simple enough for use across sites while providing sufficient information about the patient to avoid mix-ups [12]. Companies, including Trakcel, Vineti, Danaher (previously, GE), are using cloud-based solutions for manufacturing ATMPs [13-15]. Although cloud-based solution systems are useful in tracking and tracing products in traditional linear logistic supply chains, data in these centralized systems are highly vulnerable to being tampered with, potentially allowing fraudulent or damaged products to enter the supply chain or data falsification. These systems focus only on the logistics part of the delivery process and fail to address the coordination issues between stakeholders (hospitals, patients, manufacturers, logistics providers, and regulatory authorities) in the advanced therapy delivery process.

As new license indications are achieved, the systems will need to be designed to change and adapt across the whole logistics supply chain. From the collection of source material to manufacturing and final delivery of therapies, there are many handoff points, creating the probabilities of mix-ups and supply chain failure. As most advanced therapies are provided as the last line of treatment for patients with critical illnesses, the failure or delay may cost them their chance of survival.

Blockchain technology is a digital ledger of transactions, agreements, and controls that are stored in a distributed network, removing the need for a centralized database. Due to its distributed and transparent nature, the data stored cannot be tampered with and is transparent, traceable, and secure [16,17]. The benefits of the blockchain technology have been explored in various sectors, including finance, health care, and agriculture [18-20]. One of the most highly discussed use cases of blockchain technology is in supply chain management. Integration of blockchain into supply chain architecture can lead to a more reliable, transparent, and resilient system [21-24]. Specific to ATMP supply chain management, a layered blockchain solution with a smart contracts business logic layer can enable a holistic way of managing various stakeholders, ensure needle-to-needle supply chain integrity, and secure data management. The decentralized nature of blockchain can allow this to happen almost instantaneously across the full ecosystem, including the ability to keep regulators updated by publishing on a public chain with zero-knowledge proofs. Cloud environments cannot provide these facilitations; and thus, require frequent on-site monitoring and auditing by regulators. The protocol of this study will focus on developing a patient-to-patient blockchain-based supply chain system for advanced therapies.

## Methods

### Description of the Blockchain-Based Supply Chain System

This study aims to create a smart and connected supply chain logistics and manufacturing management platform based on blockchain, with cell and gene therapy as a use case. The data collected from sensors can be better connected using an interactive platform to improve coordination between stakeholders. To ensure the security of this platform, which contains sensitive information such as patient data and manufacturing protocols, blockchain allows a transparent, incorruptible, yet encrypted way of tracking and tracing the environmental conditions of advanced therapies from their origins to final administrations. This is particularly important for advanced therapies due to their requirements of cold chain delivery and the sensitivity of live cell products to fluctuations in the environment. The platform technology will allow real-time and centralized monitoring of the needle-to-needle process without compromising patient data security through selective disclosure of information.

Publishing zero-knowledge proofs, when all steps are completed as per the standard protocol, can allow regulatory authorities and manufacturers with multiple collection centers and manufacturing facilities to manage compliance by exception instead of looking through all data points and paper batch records to find regulatory breaches, allowing a reduction in regulatory monitoring costs. This can help improve consistency in all steps of collection, manufacturing, and delivery across centers and allow issues to be identified and the process to be improved through learning from data. The system will adhere to the EU and US good manufacturing practices and industry bodies for health care distribution requirements.

### Development and Evaluation

Based on the agile framework [25], this study will involve the following components for the development and evaluation of the system.

1. Stakeholder analysis
2. Customize and implement track-and-trace software platform
3. Migration and integration of software to blockchain platform
4. Case study for evaluation of the system

#### Stakeholder Analysis

A detailed stakeholder map shall be derived, including the manufacturer of therapies, health care professionals who engage with the therapy delivery pathway, payers of the treatment (for visibility of the chain of custody of the ATMP), the advanced therapy treatment center, hospital IT system implementation partner, supply chain track-and-trace provider, logistics provider, manufacturing facilities (including contract manufacturers), and regulatory authorities. Focus group meetings will be held to understand the points where regulatory/monitoring costs arise; how a network of facilities is currently managed; the data that need to be collected at different points of the supply chain to enable cheaper monitoring and product release by exception. Outcomes will be user requirement specification for decentralized manufacturing, standardized workflow for target therapy, and the understanding of the capability and gaps in and across the current systems.

#### Customize Track-and-Trace Software Platform

The design of minimum viable ecosystem and definition of scope of work for each party will be produced. A working prototype of the track-and-trace software platform for autologous advanced therapies has been developed and will be customized to cell and gene therapy. For regulatory activities, we will extend the existing regulatory approval to complete preliminary pathways for support of our new product. The platform will be designed so that it can be easily integrated into existing commercial applications, or it can be a standalone solution if required. A customized prototype will be developed to demonstrate the platform.

#### Migration and Integration of Software to Blockchain Platform

The prototype track-and-trace software platform will be adapted to combine the characteristics of linear and circular supply chains for advanced therapies. Key outcomes to monitor will be communication time reduction, capacity utilization, resource sharing and utilization, ease of establishing a process comparability across manufacturing facilities, delivery accuracy and reduced number of mix-ups/failures, user satisfaction (eg, ease of use and interoperability).

The core codes of blockchain technology platform for solutions currently developed are patented in the United States and the European Union [26]. Compared to the existing local electronic batch record systems and cloud-based solutions, a blockchain-based platform will allow for more secure ways of recording and storing supply chain data on a distributed ledger and encrypting those data to make the system tamper-proof. Any divergence or discrepancy to the ledger of information captured will be flagged to all, including the authorities.

Participating parties can manage the system so that only the relevant information is made available to each party. A blockchain solution facilitates partnerships between parties with divergent commercial self-interests, leveraging automated smart contracts. Each party is kept honest as the collective objectives of all consortium members using the blockchain solution will not be beneficial to any of the members if members were to collude. Real-time and up-to-date manufacturing process updates are transparent for patients and hospital staff in order to allow more efficient scheduling and resource allocation for treatment preparation. This means that the hospital can provide patient status updates to manufacturing. Information on the blockchain can be encrypted and made selectively available to the approved parties, hence reducing the number of GDPR-trained staff, thus lowering the cost of compliance.

Through integrating the track-and-trace and manufacturing execution system, a greater coordination between stakeholders can be fostered, allowing the delivery process to be more efficient and releasing valuable time of skilled workers for value-added work. Good track-and-trace documentation will allow the manufacturers and hospitals to review their process outcomes to improve the quality and consistency of raw material collection and manufacturing processes.

Use of blockchain will ensure data integrity between the various systems, scanners, and data input devices without mandating substantial integration changes. Moreover, blockchain creates efficiencies through automating payment processes, regulatory reporting, and compliance and audit. This technology can lower the cost of regulatory compliance through publishing zero-knowledge proofs where all standard protocols are followed properly, removing the need for going through all records (on paper or in a cloud). Furthermore, it reduces the cost of GDPR compliance training through encryption of patient data and implementation of multiple levels of access rights.

#### Case Study

This investigation will be structured via a 6-stage process for case study investigations [27] to evaluate the system’s feasibility and economic impact (Table 1). A case study will provide a structured means to generate evidence to subsequently evaluate such claims by collecting baseline data for further evaluation. It will provide an understanding of the feasibility and economic impacts of blockchain for cell therapy in a decentralized fashion at different levels of automation and at different demand levels and scales of the system. The project outcomes and appraisals will help simulate the impacts of widespread use of this methodology for larger systems through modeling. The results will be disseminated through publications and conference presentations.

**Table 1:** Case study framework.

Stage	Outcome
Plan	Case description and linking of case approach to investigation outcomes.
Design	Construction of research design and linkage of research questions, data, and criteria for evaluation and synthesis.
Prepare	Drafting, execution, and approval of study protocols.
Collect	Data collection strategy.
Analyze	Extraction of data into categories for review and analysis.
Share	Publication of the findings in a peer-reviewed journal.

## Results

This proposed study protocol will lay the foundation for future grant applications, and its execution will be completed in 2021.

## Discussion

The successful implementation of the integrated blockchain solution to the supply chain and manufacturing of advanced therapies can push the industry standards toward a safer and more secure therapy delivery process and encourage rapid adoption of the innovative technology. Adoption of the innovation in a wider context can reduce the costs of monitoring good manufacturing practices through increased transparency and security of the manufacturing and delivery process. A more transparent process can reduce communication errors and overall time spent, hence reducing health care costs. The integrated platform solution will enable more accurate and transparent tracking at all stages of the needle-to-needle delivery pipeline, allowing treatment-centric care pathways to be truly personalized through a digital evolution in supply chain and manufacturing management.

## Abbreviations

ATMP: advanced therapy medicinal products
GDPR: General Data Protection Regulation
HIPAA: Health Insurance Portability and Accountability Act
IT: information technology
